# Mixed Methods in CAM Research: A Systematic Review of Studies Published in 2012

**DOI:** 10.1155/2013/187365

**Published:** 2013-12-22

**Authors:** Felicity L. Bishop, Michelle M. Holmes

**Affiliations:** ^1^Centre for Applications of Health Psychology, Faculty of Social and Human Sciences, University of Southampton, Southampton SO17 1BJ, UK; ^2^School of Life Sciences, University of Westminster, London W1W 6UW, UK

## Abstract

*Background*. Mixed methods research uses qualitative and quantitative methods together in a single study or a series of related studies. *Objectives*. To review the prevalence and quality of mixed methods studies in complementary medicine. *Methods*. All studies published in the top 10 integrative and complementary medicine journals in 2012 were screened. The quality of mixed methods studies was appraised using a published tool designed for mixed methods studies. *Results*. 4% of papers (95 out of 2349) reported mixed methods studies, 80 of which met criteria for applying the quality appraisal tool. The most popular formal mixed methods design was triangulation (used by 74% of studies), followed by embedded (14%), sequential explanatory (8%), and finally sequential exploratory (5%). Quantitative components were generally of higher quality than qualitative components; when quantitative components involved RCTs they were of particularly high quality. Common methodological limitations were identified. Most strikingly, none of the 80 mixed methods studies addressed the philosophical tensions inherent in mixing qualitative and quantitative methods. *Conclusions and Implications*. The quality of mixed methods research in CAM can be enhanced by addressing philosophical tensions and improving reporting of (a) analytic methods and reflexivity (in qualitative components) and (b) sampling and recruitment-related procedures (in all components).

## 1. Introduction

Quantitative research methods have long dominated conventional medical research: randomised clinical trials (RCTs) produce the highest levels of evidence and occupy the pinnacle of methods hierarchies (e.g., [1]). In complementary and alternative medicine (CAM) research RCTs and methods hierarchies are more contentious, not least due to perceived fundamental differences between CAM and biomedicine [2, 3]. Unlike new pharmaceutical therapies CAM is already being provided, practiced, and used in the community; many CAMs are best conceptualized as multifactorial complex interventions; CAMs can have diverse and unanticipated effects on people; and the underlying mechanisms of many CAMs are still being researched [2, 3]. These characteristics of CAM (a) make it difficult to design good clinical trials and (b) emphasise the need to understand patients' perspectives. CAM methodologists have thus called for greater plurality in research methods in general and more use of qualitative research methods in particular [4–7].

Mixed methods research uses qualitative and quantitative approaches (or “components”) in a single study or a series of related studies. Combining qualitative and quantitative approaches permits researchers to address a wider range of research questions, to meet the diverse needs of different stakeholders, to realise the complementary strengths of both approaches, and to produce more comprehensive accounts of complex phenomena. While the idea of mixed methods research may have intuitive appeal, the methodological issues are not necessarily obvious. Creswell and Clark explain how mixed methods research can be considered from a philosophical perspective (as a methodology) and a technical perspective (as a method):

 “As a methodology, it [mixed methods research] involves philosophical assumptions that guide the direction of the collection and analysis of data and the mixture of qualitative and quantitative approaches in many phases in the research process. As a method, it focuses on collecting, analysing, and mixing both quantitative and qualitative data in a single study or series of studies” [8, p.5].

The technical perspective focuses on the practicalities of combining quantitative techniques (e.g., physiological measurement) with qualitative techniques (e.g., in-depth interviews). While undoubtedly important, the technical perspective alone is insufficient: mixed methods should also be viewed from a philosophical perspective in order to fully realise its benefits and avoid its pitfalls. This is because qualitative and quantitative approaches are typically associated with and judged according to different sets of underlying assumptions and priorities (characterized in Table 1).

At the most fundamental level, quantitative research is characterized by a (realist) belief in an independent reality which is knowable. Qualitative research is characterized by a (relativist) belief that the world is only knowable through our conceptual frameworks, which may differ between individuals and cultures. These extreme ontological positions are incommensurable—there cannot both be an independent, external reality and a reality that only exists as we apprehend it through our conceptual frameworks. Whether or not there is an independent reality is unprovable and does not have a meaningful impact on how we go about ascertaining the nature of that reality. Epistemological positions, however, can have a meaningful impact on methodology and if one ignores epistemological issues then one risks producing poor quality research [9–11]. Often epistemological issues are implicit, particularly in quantitative research, but it is important to explicitly consider them when using mixed methods. A researcher who is guided by a quantitative epistemology when conducting a qualitative interview study would attempt to collect a large representative sample of participants and use a rigid structured approach to data collection and analysis: they would thus fail to capture the richness and social and idiosyncratic nuances that qualitative data can reveal. A researcher who is guided by a qualitative epistemology when conducting a clinical trial would collect a small unrepresentative sample and leave important sources of bias uncontrolled: they would thus fail to make the robust causal inferences that are afforded by good trial design. Rigorous mixed methods research therefore requires both technical and philosophical strategies to address the challenges of combining qualitative and quantitative approaches (for details of such strategies see [8, 12–15]).

Previous reviews have described the prevalence and quality of mixed methods studies in health services research [16–18], social and behavioural sciences [19, 20], and various biomedical disciplines [21–23]. The purpose of this review was to provide a critical overview of the current state of mixed methods research in CAM. The objectives were to describe major mixed methods designs and their use in CAM research; to identify the strengths and limitations of mixed methods studies published in CAM journals; and to suggest strategies to improve the design, conduct, and reporting of future mixed methods CAM research.

## 2. Materials and Methods

### 2.1. Identifying Mixed Methods Studies

All mixed methods studies published in leading CAM journals during 2012 were eligible for inclusion in this review. The search was limited to publications in 2012 to ensure a focus on current mixed methods research. The top 10 journals in the web of knowledge category “integrative and complementary medicine” were selected in descending order of 2012 impact factor: Evidence-based Complementary and Alternative Medicine (e-CAM), Alternative Medicine Review, Phytomedicine, Journal of Ethnopharmacology, BMC Complementary Alternative Medicine, Integrative Cancer Therapies, American Journal of Chinese Medicine, Complementary Therapies in Medicine, Forschende Komplementärmedizin/Research in Complementary Medicine, and the Journal of Alternative and Complementary Medicine. Journals were selected based on impact factor as these are the most cited CAM journals: this suggests they are the most widely read and have the most influence on the scientific literature. Initially, all titles and abstracts of research papers published in these journals in 2012 were reviewed against a working definition of mixed methods research as research that collects and analyses quantitative and qualitative data [8] (see the appendix). Full texts were obtained and reviewed when it was not possible to ascertain the study design from the title and abstract alone. One researcher (MH) classified all articles in this way; a second (FB) checked 10% of articles for accuracy; discrepancies were resolved through discussion. A conservative approach was taken in that when an abstract and/or title suggested mixed methods might have been used, the full text of the article was obtained and screened. In total, 95 articles were identified as reporting mixed methods studies. Ten of the 95 articles reported on studies using a single method (e.g., quantitative) but described how this related to another study which used a different method (e.g., qualitative). In nine of these cases it was possible to locate the article reporting the related study and each pair of articles was included as one study in subsequent analyses. Finally, the studies were screened for inclusion based on the criteria set out in the quality appraisal tool described below.

### 2.2. Quality Appraisal Methods

Quality criteria differ for qualitative and quantitative methods, because they have different underlying assumptions and aims. Studies using different methods are therefore typically subject to quality appraisal using different tools (e.g., [24–30]). These tools cannot be easily adapted to assess mixed methods studies and so new tools have been developed for systematic reviews which include both qualitative and quantitative studies or which review mixed methods studies per se [31, 32]. For this review the Mixed Methods Appraisal Tool (MMAT) [33] was chosen because it was developed systematically [31] and can be used quickly and reliably [34] and it has separate subsets of items appraising the quality of (1) qualitative methods, (2) quantitative methods (using different criteria for different types of quantitative component), and (3) mixed methods (i.e., the approach to combining qualitative and quantitative components).

The MMAT [33] consists of six subsets of items as follows: screening (two items assessing whether the studies do indeed have a mixed methods research question and report relevant data to allow further quality appraisal); qualitative (four items assessing the quality of the qualitative component); quantitative RCTs (four items assessing the quality of the quantitative component if it has a randomised controlled design); quantitative non-randomised (four items assessing the quality of the quantitative component if it has more than one group but no randomisation); quantitative descriptive (four items assessing the quality of the quantitative component if it has a descriptive design such as a case study or survey); mixed methods (three items assessing the quality of the particular combination of qualitative and quantitative components). Items are worded to reflect good quality (e.g., “Is there a clear description of the randomization?”) and each study is rated as “yes,” “no,” or “cannot tell” for each applicable item. Because the MMAT items reflect quality of reporting as well as quality of study design, no attempt was made to obtain further details about the studies under review by contacting authors.

Two researchers familiarised themselves with the MMAT by studying the tutorial [33] before applying the MMAT to mixed methods CAM studies. The mixed methods design and the design of each qualitative and quantitative component was also recorded, using the definitions supplied on the MMAT. To ensure this review attended to mixed methods at a philosophical level as well as a technical level each study was also examined for the provision of any statement about how the philosophical challenges of mixed methods had been identified and/or resolved. All ratings were entered into a spread sheet. MH conducted the initial appraisal. FB then reviewed all studies, checking the results of the initial appraisal for accuracy against the full text publications. Disagreements were resolved through discussion and consensus was reached on all ratings.

**Figure 1:**
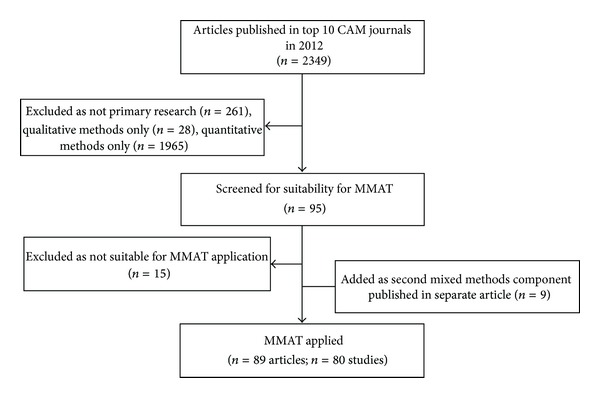
Selection of articles and studies for review.

Following application of the MMAT screening questions, three articles did not meet the criteria for full application of the MMAT and 12 articles were identified that reported ethnopharmacological/ethnobotanical studies in which the quantitative component was a laboratory-based quantitative study of plants. The MMAT does not have an appropriate section for appraising studies which do not involve human participants, so these studies were also excluded at this stage. The full MMAT was applied to 80 studies [35–114]; nine of the 80 reported their second mixed methods component in separate publications [115–123].  Figure 1 summarises the selection and screening of articles for inclusion.

## 3. Results and Discussion

### 3.1. Results

#### 3.1.1. Prevalence of Mixed Methods Studies

**Figure 2:**
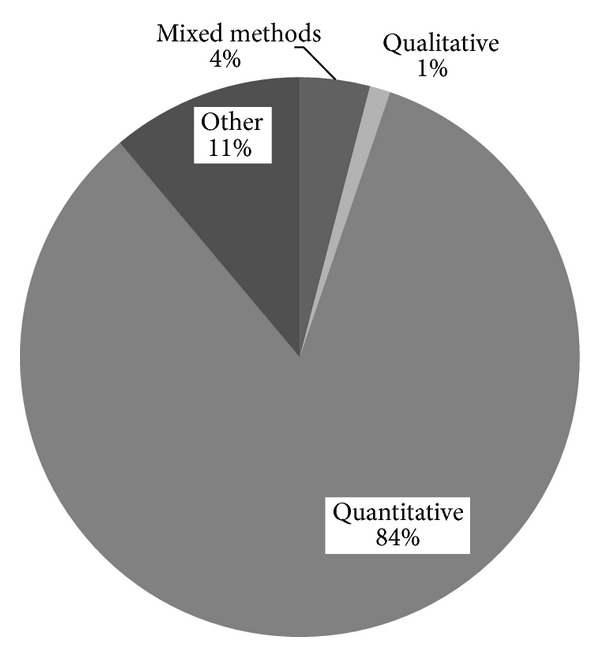
Use of different methods in studies published in the top 10 CAM journals in 2012.

As Figure 2 shows, quantitative studies dominated the top 10 CAM journals in 2012, representing 84% of all published papers. Only 4% of papers reported mixed methods studies while just 1% reported studies using qualitative methods alone. The other 11% of papers did not report primary research (these included protocols, reviews, and theoretical pieces).

#### 3.1.2. Study Designs

Of the mixed methods studies, the most common study design for qualitative components was ethnography, used by 52 studies (65%). Qualitative components also used phenomenological methods (*n* = 19, 24%), qualitative description (*n* = 5, 6%), grounded theory (*n* = 2, 2.5%), and qualitative case study (*n* = 2, 2.5%). The most common study design for quantitative components was an incidence or prevalence study (*n* = 58, 72.5%), followed by RCT (*n* = 10, 12.5%), cross-sectional analytical study (*n* = 5, 6%), case report (*n* = 3, 4%), cohort study (*n* = 2, 2.5%), and case series (*n* = 2, 2.5%).

**Figure 3:**
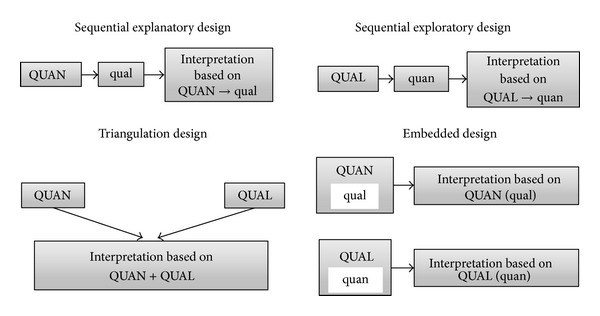
Illustration of four major mixed methods designs. Key: QUAN indicates quantitative component; QUAL indicates qualitative component. CAPITALS indicate component is typically emphasised or prioritised in this design. Lower case indicates component is typically used in a supportive capacity. Based on Creswell and Clark [8].

The MMAT distinguishes between four major mixed methods designs: sequential explanatory, sequential exploratory, triangulation, and embedded. These designs are illustrated in Figure 3 and are described in detail by Creswell and Clark [8].

In sequential mixed methods designs, the qualitative and quantitative components are conducted one after the other. In sequential explanatory designs the quantitative component is first and is used to guide the qualitative sampling; the qualitative component is then undertaken in an attempt to explain the quantitative results. Six studies in this review (8%) used a sequential explanatory design, four of which undertook a broadly phenomenological qualitative study with a subsample of participants from an RCT.

In sequential exploratory designs, the qualitative component is undertaken first to explore a research area; the quantitative component is then used to extend or generalize the qualitative results. Four studies in this review (5%) used a sequential exploratory design, all of which aimed to develop or refine a questionnaire tool.

In triangulation and embedded mixed methods designs, the qualitative and quantitative components are concomitant. The purpose of triangulation designs is to examine the same phenomenon from multiple perspectives; the qualitative and quantitative components can involve the same participants, can be analysed together (by transforming qualitative data into quantitative data or vice versa), or can be brought together after analysis and during interpretation. The triangulation design was the most common in this review, used by 59 studies (74%). Many of these were ethnopharmacological studies which used ethnographic methods in their qualitative component and incidence or prevalence studies in their quantitative component.

Embedded designs typically involve the same participants in each component and prioritise one component over the other; the latter is then used in a supportive capacity. Eleven studies in this review (14%) used an embedded design, all of which embedded a qualitative component within a major quantitative component.

#### 3.1.3. Quality Appraisal

The MMAT results suggest that some mixed methods elements were of good quality and the quantitative components were generally of higher quality than the qualitative components (Table 2). In the majority of studies the mixed methods design was relevant to the research questions and the qualitative and quantitative components were integrated at some stage to address the research question. Often this integration occurred at the interpretation stage but sometimes it occurred during data analysis. Only 5% of studies acknowledged or reflected on the limitations of their mixed methods design and none of the studies acknowledged or addressed the philosophical tensions involved in mixed methods research.

In terms of the individual components, quantitative RCT components scored especially highly: all RCTs reported at least 80% outcome data and the majority had low dropout rates and clearly described appropriate randomization and allocation concealment procedures. Non-randomised quantitative components were also generally of high quality, particularly regarding the validity of measurements and the use of recruitment procedures to minimise selection bias. The majority of descriptive quantitative components also used appropriate and valid measurements. Common weaknesses were identified in the qualitative components. The majority of qualitative components did not give appropriate consideration to the impact of the researchers or the wider context on the methods/findings (i.e., there was no evidence of reflexivity and very little description of the researchers themselves or the research settings).

The frequency of “cannot tell” ratings was particularly high for some MMAT items. For the qualitative components, there was often insufficient detail to evaluate the sampling and data analysis procedures. Details about sampling were also lacking in non-randomised and descriptive quantitative components. The majority of quantitative descriptive components failed to report a response rate. A large minority of quantitative non-randomised components either did not report baseline comparisons between groups or failed to control for relevant confounders.

### 3.2. Discussion

Mixed methods studies represent 4% of papers published in the top 10 CAM journals in 2012. Some reviews have also found low numbers of mixed methods studies in primary care [22], mental health nursing [21], and health services research [18]. In comparison, other reviews suggest mixed methods are used more often: one review of UK-funded health services research found that 18% of studies are mixed methods [17]; nursing and education journals publish more mixed methods studies (16%) than psychology and sociology journals (6%) [19].

Among the 80 studies included in this review the most popular mixed methods design was triangulation: qualitative and quantitative components were conducted concomitantly (with the same or different participants) and brought together to address the research question(s). A few studies used embedded designs in which qualitative and quantitative components were not only concomitant but also typically involved the same participants and prioritised one component over the other. A minority of mixed methods studies conducted qualitative and quantitative components sequentially, with similar numbers of studies using explanatory (quantitative component first) and exploratory (qualitative component first) designs. In health services research concomitant designs are also somewhat more popular than sequential designs [17]. This might be because concomitant designs appear to be more efficient than sequential designs and can be completed more quickly, although they can also be more intensive as the two components are undertaken at the same time and it can be more difficult for one component to build on and incorporate insights from the other.

The reporting related to mixed methods was of mixed quality. In most studies, the mixed methods design and integration of results were appropriate to the research question(s). However, very few studies reflected on the limitations of their mixed methods design and none addressed the philosophical tensions inherent in mixed methods research. Possible reasons for the lack of discussion of philosophical issues include lack of awareness, deliberate omission, and insufficient space for reporting. In future, CAM researchers might consider pragmatism as a way of addressing the philosophical challenges of mixed methods research [8, 15]. A full discussion of pragmatism is beyond the scope of this paper, but in essence a pragmatic perspective involves judging a piece of research on the extent to which it achieves its stated external goals [12]. Applying pragmatism, a qualitative component might be judged on the extent to which the findings enable an intervention to be delivered in a way that respects and engages patients; a quantitative component might be judged on the extent to which it provides persuasive evidence that leads to policy makers funding an intervention. This perspective encourages researchers to engage with qualitative and quantitative approaches as methodologies and thus make the most of their complementary strengths. For examples of mixed methods CAM research which uses a pragmatic perspective see [12, 124].

Among the CAM mixed methods studies reviewed, the quantitative components were generally of higher quality than the qualitative components. This reflects how most of the studies prioritised quantitative components over qualitative components. It may also indicate greater expertise among the CAM research community in quantitative methods or publishing bias in favour of quantitative studies, both of which would be consistent with the overall dominance of quantitative studies in CAM journals. More limited reporting of qualitative components was also noted in a review of mixed methods studies in health services research [16].

Limitations of this review must be acknowledged. Mixed methods CAM studies published in non-CAM journals (e.g., mainstream medical, health services research, and social science) or in CAM journals with lower impact factors were not included. The results provide a snapshot of studies published in 2012 that may not reflect the situation before or since. By focusing on published articles (rather than also searching for protocols), studies that were designed as mixed methods but written up independently will have been overlooked: space restrictions in print journals may contribute to this phenomena, but as online journals become more popular space restrictions should become less of a barrier to publishing mixed methods research. The majority of mixed methods studies included in this review were ethnopharmacological studies, many of which scored poorly on the MMAT because they provided few details about sampling in general and qualitative analysis methods in particular. This may reflect different reporting standards within ethnopharmacology compared to other CAM disciplines. The MMAT was not suitable for assessing mixed methods studies in which the quantitative component involved laboratory work: to include such studies in future mixed methods reviews would require an alternative quality appraisal tool.

## 4. Conclusions

Mixed methods studies are being conducted by CAM researchers and reported in CAM journals, but they represent a very small proportion of all published studies. The quality of mixed methods research in CAM can be improved if researchers

1. attend to the philosophical tensions inherent in mixing qualitative and quantitative methodologies and reflect on the limitations and challenges of the chosen mixed methods design,
2. report on sampling strategies, participation rates, and related issues in more detail (in both qualitative and quantitative components),
3. explicitly consider the impact of the researcher and the broader context on the findings and report data analysis methods transparently and in detail (in qualitative components).

By addressing these points, the standard of mixed methods research in CAM can be improved and the benefits of this approach can be more fully realised.

## Figures and Tables

**Table 1:** Simplified comparison of typical characteristics of quantitative and qualitative approaches to research.

Characteristic	Quantitative approaches	Qualitative approaches
Ontology	Realist	Relativist
Epistemology	Knowledge limited only by technologies of knowing	Knowledge is embedded in value and culture (including the research process)
Aims/intended outcome	Universal laws	Locally situated and contextualised understandings
Relationship between researcher and participants	Distant, objective	Close, subjective
Scope	General, nomothetic	Specific, idiographic
Nature of information	Causal, mechanistic explanation and prediction	Meaning, understanding
Relationship between theory and data	Hypothetico-deductive-data confirms/falsifies theory	Inductive—theory emerges from data

**Table 2:** Results of quality appraisal of 80 mixed methods studies using MMAT [33].

	Yes		No		Cannot tell	
	*n*	%	*n*	%	*n*	%
*Qualitative Component *						
(1.1) Are the sources of qualitative data relevant to address the research question?	35	43.8%	2	2.5%	43	53.8%
(1.2) Is the process for analyzing qualitative data relevant to address the research question?	44	55.0%	3	3.8%	33	41.3%
(1.3) Is appropriate consideration given to how findings relate to the context in which the data were collected?	30	37.5%	50	62.5%	0	0.0%
(1.4) Is appropriate consideration given to how findings relate to researchers' influence?	17	21.3%	63	78.8%	0	0.0%

*Quantitative Component—RCTs *						
(2.1) Is there a clear description of the randomization (or an appropriate sequence generation)?	8	80.0%	2	20.0%	0	0.0%
(2.2) Is there a clear description of the allocation concealment (or blinding when applicable)?	7	70.0%	3	30.0%	0	0.0%
(2.3) Are there complete outcome data (80% or above)?	10	100.0%	0	0.0%	0	0.0%
(2.4) Is there low withdrawal/dropout (below 20%)?	8	80.0%	2	20.0%	0	0.0%

*Quantitative Component—Nonrandomised *						
(3.1) Are participants (organizations) recruited in a way that minimizes selection bias?	5	71.4%	0	0.0%	2	28.6%
(3.2) Are measurements appropriate regarding the exposure/intervention and outcomes?	7	100.0%	0	0.0%	0	0.0%
(3.3) In the groups being compared, are the participants comparable, or do researchers take into account (control for) the difference between these groups?	4	57.1%	0	0.0%	3	42.9%
(3.4) Are there complete outcome data (80% or above) and, when applicable, an acceptable response rate (60% or above) or an acceptable follow-up rate for cohort studies (depending on duration of followup)?	4	57.1%	2	28.6%	1	14.3%

*Quantitative Component—Descriptive *						
(4.1) Is the sampling strategy relevant to address the quantitative research question?	18	28.6%	2	3.2%	43	68.3%
(4.2) Is the sample representative of the population understudy?	12	19.0%	2	3.2%	49	77.8%
(4.3) Are measurements appropriate (clear origin, or validity known, or standard instrument)?	51	81.0%	8	12.7%	4	6.3%
(4.4) Is there an acceptable response rate (60% or above)?	7	11.1%	6	9.5%	50	79.4%

*Mixed Methods *						
(5.1) Is the mixed methods research design relevant to address the qualitative and quantitative research questions?	64	80.0%	12	15.0%	4	5.0%
(5.2) Is the integration of qualitative and quantitative data (or results) relevant to address the research question?	65	81.3%	10	12.5%	5	6.3%
(5.3) Is appropriate consideration given to the limitations associated with this integration?	4	5.0%	76	95.0%	0	0.0%

## Appendix

### Working Definitions Used to Code Abstracts for Methods

*Quantitative Studies: Collect Quantitative Data and Analyse It Quantitatively*. Quantitative data are numerical. They include answers to questionnaires where the respondent has fixed response options, whether these response options have verbal labels (e.g., yes/no; strongly agree, agree disagree, strongly disagree) or numeric labels (e.g., 1, 2, 3, 4, 5). They also include more “objective” measures, for example, biological, chemical, or physiological measures, EEGs, MRIs, reaction times, and so forth. Quantitative analyses involve manipulating numbers. This might be as simple as reporting the frequency of particular events. More often, quantitative analyses will involve some kind of statistical calculations. Studies which use an experimental design (e.g., psychology experiments and clinical trials) are typically (but not always) quantitative. Studies which use questionnaires may be quantitative but may also be qualitative. A quantitative questionnaire study that has only very simple and short open-ended questions of the type “other, please specify” should be classified as quantitative.

*Qualitative Studies: Collect Qualitative Data and Analyse It Qualitatively*. Qualitative data are not numerical. They are typically verbal or textual, that is, involve words. Qualitative data can involve spoken words or written words or visual data (e.g., photos and videos). Qualitative data are typically collected via open-ended questions on a questionnaire, via semistructured interviews (or other types of interviews, e.g., “depth” “unstructured” “narrative”), or via focus groups or other group discussions. Qualitative data might also be collected through recording naturalistic events (e.g., audio or video recording naturally occurring conversations, medical consultations, etc.), by collecting documents (e.g., newspaper articles and policy documents) or by eliciting documents (e.g., asking people to write diaries or take photos). Qualitative analyses involve describing or interpreting qualitative data, without converting the data into numbers. For example, this might involve classifying talk into categories or themes and then describing those themes. Some phrases that might be used to describe qualitative analysis include thematic analysis, grounded theory, IPA, phenomenological analysis, framework, framework analysis, discourse analysis, and conversation analysis.

*Mixed Methods Studies: “Mixed Methods Research Involves Both Collecting and Analysing Quantitative and Qualitative Data” [8]*. Mixed methods studies involve some quantitative data collection and some qualitative data collection. It is possible for the qualitative data to be converted into quantitative data during and/or in preparation for analysis (or vice versa). This still counts as mixed methods. Qualitative studies that are “nested within” or “embedded in” quantitative studies also count as mixed methods, for example, a qualitative study where the participants are recruited because they took part in a specific clinical trial—this would count as mixed methods, even if the results of the trial are not reported in the same paper as the results of the qualitative study.
